# Funding the critical path from research to reality: sustaining HIV vaccine R&D amidst economic uncertainty

**DOI:** 10.1186/1742-4690-9-S2-P236

**Published:** 2012-09-13

**Authors:** K Fisher, ED Donaldson, B Gobet, L Green, T Harmon, P Harrison, R Lande, M Warren

**Affiliations:** 1 AVAC, New York, NY, USA; 2 UNAIDS, The Joint United Nations Programme on HIV/AIDS, Geneva, Switzerland; 3 International Partnership for Microbicides, Silver Spring, MD, USA; 4 International AIDS Vaccine Initiative, New York, NY, USA

## Background

Since 2004, the HIV Vaccines and Microbicides Resource Tracking Working Group has employed a comprehensive methodology to track trends in R&D investments and expenditures for biomedical HIV prevention options, including HIV vaccines, microbicides, pre-exposure prophylaxis (PrEP), treatment as prevention, adult voluntary medical male circumcision, female condoms, HSV-2 prevention and vertical transmission prevention. Such monitoring is critical to identifying trends in investment and providing a fact base for policy advocacy on R&D spending levels and allocations.

## Methods

To estimate annual investment in HIV vaccine R&D, data were collected from government agencies, nonprofit research organizations, foundations and pharmaceutical/biotechnology companies on annual disbursements for product development, clinical trials and trial preparation, community education and policy advocacy efforts.

## Results

Preliminary estimates suggest that public and philanthropic funding for HIV vaccine research has been experiencing increased stress. As funding priorities shifted within governments and major donors revised their R&D grant strategies, disbursements for research in 2011 were often flat-lined or decreased. While the biotechnology industry continued their R&D efforts, companies increasingly focused on forming product development partnerships to ensure future funding and relied on grants from public sector sources to continue their clinical trials.

## Conclusion

2011 saw continued progress toward the development of a safe and effective HIV vaccine. With the discovery of antibodies predicting the risk of HIV infection, the start of large-scale follow-on trials and the initiation of HIV vaccine R&D programs in more countries around the world, there has been real progress on the path from research to reality. However, funding to pursue all this promise is highly uncertain. Sustained resources will be essential to assure that trials now underway can proceed, and increased expenditures will be similarly essential for the successive clinical phases of the most promising vaccine candidates.

